# Moral Injury in Trauma-Exposed, Treatment-Seeking Police Officers and Military Veterans: Latent Class Analysis

**DOI:** 10.3389/fpsyt.2022.904659

**Published:** 2022-07-11

**Authors:** Beijka Mensink, Annette van Schagen, Niels van der Aa, F. Jackie June ter Heide

**Affiliations:** ARQ Nationaal Psychotrauma Centrum, ARQ Centrum'45, Oegstgeest, Netherlands

**Keywords:** moral injury, post-traumatic stress disorder, occupational trauma, uniformed personnel, police officers, military veterans

## Abstract

Exposure to morally injurious events may have a severe, prolonged negative impact on psychosocial functioning, known as moral injury (MI). Research into the prevalence of MI has mostly focused on event exposure rather than on psychosocial impact. Also, the relationship between MI and post-traumatic stress disorder (PTSD) remains a matter of interest. The aim of this study was to identify MI and PTSD symptom profiles among trauma-exposed, treatment-seeking police officers and military veterans, and to explore demographic and clinical differences between symptom profiles. Latent class and multinomial regression analyses were conducted in a sample of 1,703 participants, using the Clinician-Administered PTSD Scale for DSM-5 and the Brief Symptom Inventory. Four classes of participants were identified, labeled as a *MI* class (*n* = 192; 11.27%), a *MI-PTSD* class (*n* = 565; 33.18%), a *PTSD* class (*n* = 644; 37.82%), and a *Neither MI-nor PTSD* class (*n* = 302; 17.73%), resulting in 44.45% (*n* = 757) of participants who met an MI symptom profile with or without PTSD. There were significant differences between the classes in terms of gender as well as PTSD and comorbid psychopathology symptom severity, the latter of which was highest in the MI-PTSD class. In conclusion, a substantial subgroup of trauma-exposed, treatment-seeking police officers and military veterans could be classified as suffering from MI. Routinely screening for MI in treatment-seeking police officers and military veterans is recommended, and interventions aimed at relieving MI in these populations may be indicated.

## Introduction

Exposure to events that take place in high stakes situations and in which deeply held moral beliefs and expectations are transgressed, may be morally injurious to those involved (1, 2). Such moral transgressions or *potentially morally injurious events* (PMIEs) include events in which a person harms another person (*commission*), a person could not prevent harm to another person (*omission*), or a person him- or herself is harmed by a person in power or authority (*betrayal trauma*). Exposure to such events may lead to severe and persistent emotional, psychological, biological, spiritual, behavioral, and social suffering, known as *moral injury* (MI*)* (1). MI centers around negative moral emotions and cognitions such as guilt, shame, anger, self-blame, existential crisis, grief, sorrow, betrayal, and distrust (3–5).

Litz et al. (1) and Litz and Kerig (6) were the first to propose a working conceptual model of MI, consisting of the following elements: (1) transgression (PMIE), leading to (2) internal dissonance and conflict with one's fundamental beliefs and assumptions, resulting in (3) stable, negative, internal global attributions about the transgression, (4) enduring moral emotions such as shame, guilt, anxiety, and anger, (5) withdrawal, (6) failure to forgive or self-condemnation, (7) self-harming and selfhandicapping behaviors and demoralization, and (8) chronic intrusions, avoidance and numbing (1, 6, 7).

Research on MI continues to advance, but the definition of what constitutes a moral transgression or PMIE, and what constitutes MI, is still a matter of discussion (e.g., (7)). This complicates research into the prevalence of both PMIEs and MI. The prevalence of PMIE exposure has been studied predominantly in military populations in the United States (8–10) and Canada (11, 12), with prevalence rates ranging from 4.8% for perpetration (9) to 65% for exposure to any PMIE (11). Research into the prevalence of MI has mostly been conducted using different versions of the Moral Injury Symptoms Scale-Military Version (MISS-M) (13). In these studies prevalence was defined either as the percentage of respondents who reported high levels of at least one symptom, ranging from 80 to 90% in United States military populations (13, 14), or as the percentage of respondents whose MI symptoms caused at least moderate impairment, ranging from 24 to 41% in Chinese health professionals (15, 16). The prevalence of PMIE exposure, and consequently of functional impairment, has been found to differ between sexes, with female veterans being at higher risk of functional impairment due to betrayal-based events and male veterans suffering more from perpetration-based events (8). In addition, a lower age has been found to correlate with higher MI scores in healthcare professionals (15, 17) and veterans with non-epileptic seizures (18).

The discussion on what constitutes MI also pertains to the relations between MI and post–traumatic stress disorder (PTSD) (19), focusing on the distinction, association, and overlap between the two concepts. A study among National Guard personnel found MI to be uniquely characterized by guilt, shame, anhedonia, anger, and social alienation, while PTSD was characterized by startle reflex, memory loss, self-reported flashbacks, nightmares, and insomnia (20). A review of the evidence suggests that PMIE exposure may lead to PTSD as well as other symptoms (such as negative moral emotions and loss of meaning) that are distinct from, but associated with PTSD (21). This discussion is at least partly related to the definition of PTSD according to DSM-5 vs. to the 11th edition of the International Classification of Diseases (ICD-11) (22). The overlap between PTSD and MI increased with the publication of a more encompassing definition of PTSD in DSM-5, which includes exaggerated negative beliefs, distorted cognitions leading to blame, persistent negative emotional state, and reckless or self-destructive behavior (19). The overlap of MI with PTSD according to ICD-11 is likely to be smaller due to the ICD-11's narrower definition of PTSD, which is limited to re-experiencing, avoidance, and sense of current threat (22).

Both the prevalence of MI and its relation with PTSD are relevant to psychological treatment. Research on treatment for MI is still limited. Given the overlap between MI and PTSD, it has been suggested that classic trauma-focused treatment such as prolonged exposure (PE) may suffice for the treatment of MI (23). However, some researchers claim that trauma-focused treatment should be adapted or supplemented with interventions specifically designed for MI, focusing on aspects that are distinct from the fear-based aspects of PTSD (e.g., (1, 21, 24)). Determining the occurrence of MI in PMIE-exposed populations may help to decide what percentage of exposed populations is in need of treatment for MI. Meanwhile, determining the separate or comorbid occurrence of MI and PTSD may help to clarify whether treatment for PTSD may suffice for those who suffer from MI, or whether additional interventions may be necessary.

To determine the occurrence of MI in PMIE-exposed populations, as well as its relationship with PTSD, we conducted a latent class analysis of MI among treatment-seeking police officers and military veterans in the Netherlands. There are no previous studies on MI in Dutch police officers, but treatment-seeking Dutch police officers are known to have been exposed to 19.5 different types of potentially traumatic events on average (25), many of which may be considered morally injurious, e.g., having to make decisions that affect the survival of others, engaging in or witnessing acts of disproportionate violence, killing or harming others in line of duty. A quarter of Dutch military veterans have been shown to experience feelings of guilt and shame after participation in peace missions, and these feelings were related to higher levels of depression and anger (26). During missions they may experience value conflicts, moral detachment, and senselessness (27).

The aim of the current study was to identify MI and PTSD symptom profiles among trauma-exposed, treatment-seeking police officers and military veterans, and to explore demographic (gender, age, and professional background) and clinical (trauma exposure, clinician-rated PTSD and self-reported general psychopathology severity) differences between classes. Given that both police officers and military veterans are exposed to PMIEs, we hypothesized that we would find a MI class among these two populations. Given that MI and PTSD have been found to be both separate and distinct, we hypothesized that we would find classes of MI with and without PTSD. Finally, based on the literature we hypothesized that we would find differences in age, gender, PTSD severity and psychopathology severity between classes. This study is a first effort and part of a larger research program aimed at assessing the validity and clinical relevance of the MI concept in treatment-seeking police officers and military veterans.

## Materials and Methods

### Design

A naturalistic, observational design was employed, utilizing routine outcome monitoring (ROM) data from pre-treatment diagnostic assessments. Data were collected at ARQ Centrum'45 and ARQ Diagnostic Centrum, two mental health partner organizations of ARQ Nationaal Psychotrauma Centrum in the Netherlands, from June 2015 to April 2021. Assessments included the Clinician-Administered PTSD Scale for DSM-5 (CAPS-5) (28, 29) and the Brief Symptom Inventory (BSI) (30, 31) to assess symptoms of PTSD and MI. Given that MI is a relatively new concept, no instruments for MI were part of the diagnostic assessment.

### Setting

ARQ Centrum'45 is a highly specialized mental healthcare institute for patients with complex and severe psychotrauma. The institute offers treatment for, among other populations, trauma-exposed police officers and military veterans who either show complex psychopathology or have not benefited from previous treatment. Treatment predominantly takes place in an outpatient setting and consists of evidence-based, trauma-focused therapy (such as Prolonged Exposure, Eye Movement Desensitization and Reprocessing, Brief Eclectic Psychotherapy for PTSD, and Narrative Exposure Therapy), combined with other forms of psychotherapy, pharmacotherapy, arts therapies, family and couples therapy and social work when indicated.

ARQ Diagnostic Centrum is a national institute for diagnostics of trauma-exposed patients, especially police officers. The institute offers diagnostic assessments only, which takes one full day and includes clinician-rated interviews and self-report measures. Patients with a (partial) PTSD diagnosis are then referred to psychotrauma therapists or institutes for treatment, including, but not limited to, ARQ Centrum'45.

### Procedure

The CAPS-5 and BSI were administered at both institutes at the initial diagnostic assessment. The CAPS-5 was administered by psychologists or psychological workers who had received a 1-day training in administering the CAPS-5 as well as regular supervision by a licensed psychologist. CAPS-5 administration took about 45–60 mins. Responses were entered into a secure digital platform for psychological assessment called QuestManager, which is linked to the patient's file. The BSI was administered through the same platform. For those patients who had a diagnostic assessment at ARQ Diagnostic Centrum and were then referred to ARQ Centrum'45, only the data of the first assessment was included in the database for this study.

Data were primarily collected for diagnostic and treatment purposes and secondarily used for research purposes. During the assessment procedure at both institutes, participants were informed about the use of anonymized ROM data for research and asked if they wished to have their data removed from the database. Upon consultation, the medical ethics committee of Leiden University stated that no review of the ethical merits of the study was needed because assessments were conducted primarily for diagnostic purposes within the institution and only secondarily for data analysis.

### Participants

The participants in this study were patients with occupational trauma related to their professional background in the police or the military, who sought treatment and were referred to either ARQ Diagnostic Centrum (for diagnostic assessment) or ARQ Centrum'45 (for treatment). Only those patients who met the A-criterion for PTSD according to the CAPS-5, and whose initial pre-treatment assessment included the CAPS-5 and the BSI, were included. Patient characteristics are described in Table 1.

**Table 1:** Demographic and clinical characteristics (*n* = 1,703).

**Characteristics**	***n***	**%**	**Mean**	**Minimum**	**Maximum**	***SD***
Age			45.48	19.71	81.80	10.75
Gender	
Male	1,264	74.30				
Female	437	25.69				
Professional background
Police forces	1,531	89.90				
Military veterans	172	10.09				
Trauma history	
Actual or threatened death	1,634	95.50				
Serious injury	1,207	70.90				
Sexual violence	134	7.90				
Setting	
ARQ Diagnostic Centrum	1,399	82.15				
ARQ Centrum'45	404	17.85				
PTSD classification	1,220	71.60				
PTSD severity (CAPS-5)			29.80	0.00	71.00	14.11
Psychopathology severity (BSI)			1.49	0.00	3.75	14.23

*PTSD, post-traumatic stress disorder; CAPS-5, Clinician-Administered PTSD Scale for DSM-5; BSI, Brief Symptom Inventory*.

The sample consisted mainly of on average middle-aged men with a professional background in the police forces, who had an assessment at ARQ Diagnostic Centrum. Exposure to actual or threatened death was the most prevalent trauma type. The majority of patients (71.6%) met the classification of PTSD according to the CAPS-5. Mean psychopathology severity as measured by the BSI fell within the above average range compared to a norm group of Dutch psychiatric outpatients.

## Measures

The Clinician-Administered PTSD Scale for DSM-5 (CAPS-5) (28) Dutch version (29) is a 30-item structured interview matching the DSM-5 classification for PTSD. Items are rated on a 5-point severity scale ranging from 0 (*absent*) to 4 (*incapacitating*). By summing the 20 symptom severity scores (Criteria B-E) a total PTSD symptom severity score is computed ranging between 0 and 80, with higher scores indicating higher severity. Psychometric evaluation has demonstrated good psychometric properties (32, 33). In the current sample Cronbach's α was 90.

The Brief Symptom Inventory (BSI) (30) Dutch version (31) is a 53-item self-report rating scale that assesses the severity of general psychopathology during the past week. Items are rated on a 5-point Likert scale ranging from 0 (*not at all*) to 4 (*extremely*). A mean severity score is calculated for the total scale (range 0–4). In comparison with a norm group of Dutch psychiatric outpatients, cut-off scores for the total scale may be interpreted as follows: 0.00–0.23 very low; 0.24–0.55 low; 0.56–0.89 below average; 0.90–1.26 average; 1.27–1.75 above average; 1.75–2.53 high; 2.54–4.00 very high (31). Good psychometric properties have been reported for the BSI (31). In the present sample Cronbach's α was 0.97.

In line with item selection methods in previous research (34, 35), items from the CAPS-5 and the BSI were used to investigate the presence of MI and PTSD symptoms. Items for MI were selected based on the working conceptual framework of Litz et al. (1), which includes the following eight elements: (1) transgression; (2) dissonance/conflict; (3) stable, internal, global attributions; (4) shame, guilt, anxiety; (5) withdrawal; (6) failure to forgive or self-condemnation; (7) self-harming, self-handicapping, demoralization; (8) chronic intrusions, avoidance, numbing. Using all the BSI items and the CAPS items D1–7 and E1–2, these elements of MI were operationalized with a set of 13 items from the BSI and eight from the CAPS-5, selected by the authors independently and compared and discussed until agreement was reached. PTSD was operationalized with a set of nine items from the CAPS-5 based on the ICD-11 diagnosis of PTSD, which includes the PTSD-symptom clusters of intrusions (items B1–5), avoidance (items C1–2), and arousal (items E3 and E4), to avoid duplication of CAPS-5 items in the MI and the PTSD subsets. Descriptions of the indicators for MI and PTSD and the matching items from the BSI and CAPS-5 used in the latent class analysis can be found in Table 2.

**Table 2:** Description of the elements of MI and PTSD and the matching items from the BSI and the CAPS-5.

	**Variable (items in latent class analysis)**	***n***	**Symptom endorsement (score ≥2)**	**%**
**Dimensions moral injury**				
MI-1: Stable, internal global attributions	BSI 10: Feeling that most people cannot be trusted	1,701	822	48.3
	BSI 22: Feeling inferior to others	1,701	660	38.8
	BSI 50: Feelings of worthlessness	1,701	627	36.9
	CAPS D2: Exaggerated negative beliefs or expectations	1,688	831	49.2
MI-2: Enduring moral emotions such as shame, guilt, anxiety and anger				
Guilt	BSI 52: Feeling of guilt	1,701	828	48.7
Shame	CAPS D3: Distorted cognitions leading to blame	1,689	426	25.2
Anxiety	BSI 19: Feeling fearful	1,701	697	41.0
Anger	BSI 13: Temper outbursts that you could not control	1,701	707	41.6
	BSI 46: Getting into frequent arguments	1,701	688	40.4
	CAPS E1: Irritable behavior and angry outbursts	1,689	1,200	71.0
MI-3: Withdrawal	BSI 14: Feeling lonely even if you are with people	1,701	921	54.1
	BSI 44: Never feeling close to another person	1,701	531	31.2
	CAPS D5: Diminished interest or participation in activities	1,688	1,177	69.7
	CAPS D6: Detachment or estrangement from others	1,688	939	55.6
MI-4: Failure to forgive or self-condemnation	BSI 34: The idea that you should be punished for your sins	1,701	155	9.1
MI-5: Numbing	CAPS D4: Persistent negative emotional state	1,689	1,305	77.3
	CAPS D7: Persistent inability to experience positive emotions	1,687	933	55.3
	BSI 18: Feeling no interest in things	1701	1110	65.3
MI-6: Self-harming and self-handicapping behaviors and demoralization	BSI 9: Thoughts of ending your life	1,701	198	11.6
	BSI 35: Feeling hopeless about the future	1,701	784	46.1
	CAPS E2: Reckless or self-destructive behavior	1,687	199	11.8
**Core symptoms of PTSD (ICD-11 definition)**				
PTSD-1: Intrusions	CAPS B1: Intrusive memories	1,698	1,314	77.4
	CAPS B2: Distressing dreams	1,696	959	56.5
	CAPS B3: Dissociative reactions	1,697	300	17.7
	CAPS B4: Cued psychological distress	1,695	1,216	71.7
	CAPS B5: Cued physiological reactions	1,692	1,220	72.1
PTSD-2: Avoidance	CAPS C1: Avoidance of memories, thoughts, feelings	1,693	1,260	74.4
	CAPS C2: Avoidance of external reminders	1,692	1,055	62.4
PTSD-3: Arousal	CAPS E3: Hypervigilance	1,689	1,124	66.5
	CAPS E4: Exaggerated startle response	1,688	672	39.8

*MI, moral injury; PTSD, post-traumatic stress disorder; CAPS-5, Clinician-Administered PTSD Scale for DSM-5; BSI, Brief Symptom Inventory; ICD-11, International Classification of Diseases-11th revision*.

### Statistical Analyses

The selected CAPS-5 and BSI items were recoded into dichotomous scores based on symptom endorsement, i.e., a cut-off value that discriminates between the presence or absence of a symptom. According to the basic CAPS-5 symptom scoring rule, a symptom is considered present if its severity is rated 2 or higher (33). A similar dichotomization rule for the BSI symptoms is not present in the literature, hence we dichotomized the BSI symptoms in a similar way as the CAPS-5 symptoms. With regard to the CAPS-5, a symptom was considered absent when it was rated as absent (severity score = 0) or mild/ subthreshold (severity score = 1) and present when it was rated as moderate/ threshold (severity score = 2), severe/ markedly elevated (severity score = 3) or extreme/ incapacitating (severity score = 4). Likewise, a BSI symptom was considered absent when the distress level was rated as not at all (0) or a little bit (1) and present when it was rated as moderate (2), quite a bit (3) or extremely (4). Latent class analysis (LCA) in Mplus version 8 (36) was used to classify participants into homogeneous latent subgroups (classes), based on similar response patterns on dichotomous symptom endorsement scores of MI and PTSD. In line with earlier LCA studies on PTSD (34, 35), a probability > 0.5 was considered as a cut-off value for symptom endorsement within the latent classes. Robust full information maximum likelihood estimation (FIMLR) was used to include participants with missing data. To avoid local likelihood maxima, 1,000 random sets of starting values in the first and 100 in the second step of optimization were requested, and 50 initial stage iterations were used. Using LCA, the minimum number of classes that can account for associations between symptoms can be identified.

We began with a one-class model and increased the number of latent classes until we achieved a model which no longer gave an acceptable fit or substantive meaning (37, 38). The most parsimonious model with acceptable model fit and classification quality, as well as theoretical meaning, was selected as the optimal solution. The following indices were used to find the optimal number of classes: Bayesian information criterion (BIC), bootstrap likelihood ratio test (BLRT), Lo-Mendell-Rubin adjusted likelihood ratio test (LMR-A), and entropy. Lower BIC and higher entropy indicate a better fit (39). For the BLRT and LMR-A, a significant *p*-value indicates that the estimated model fits the data better than the model with one less class (40). To avoid local likelihood maxima in BLRT, 500 bootstrap samples were requested with 50 sets of starting values in the first and 20 in each bootstrap sample. The entropy statistic was used to evaluate the overall quality of classification, which is considered adequate when entropy values are >0.80 (41). The most likely class membership for the participants was derived from the optimal latent class model.

Whether the covariates of age, gender, pre-treatment assessment of PTSD severity (CAPS-5), and comorbid psychopathology severity (BSI) differentiated between the latent class representing MI and the other classes, was tested by conducting a series of multinomial logistic regression models using the three-step procedure in Mplus (42). Because data on the covariates were available for subsamples of different sizes and because Mplus handles missing values in the covariates with listwise deletion, separate multinomial regression models were estimated. Age and gender were tested in one model. PTSD severity and severity of comorbid psychopathology were each tested in a separate multinomial regression model. The latter was also done to check for possible interference by CAPS-5 and BSI items included as an indicator in the LCA, as well as being part of the PTSD severity score and comorbid psychopathology score in the multinomial logistic regression part of the model.

## Results

### Latent Class Analysis

Model fitting results of the seven models with one- to seven-class solutions are presented in Table 3.

**Table 3:** Model fitting results of the seven models with one- to seven-class solutions.

**Model**	**Log-likelihood**	**BIC**	**BLRT**		**LMR-A**		**Entropy**
			**-2LL difference**	***p*-value**	**Value**	***p*-value**	
1 class	−30,875.614	61,974.432	–	–	–	–	1.000
2 classes	−27,387.325	55,228.499	6,976.577	0.000	6,946.460	0.000	0.876
3 classes	−26,339.465	53,363.424	2,095.719	0.000	2,086.672	0.000	0.875
**4 classes**	**−25,795.780**	**52,506.698**	**1,087.370**	**0.000**	**1,082.676**	**0.000**	**0.887**
5 classes	−25,522.575	52,190.932	546.411	0.000	544.052	0.000	0.853
6 classes	−25,379.389	52,135.206	286.370	0.000	285.134	0.0402	0.862
7 classes	−25,235.762	52,078.597	287.254	0.000	286.014	0.1153	0.840

*Most meaningful model is printed in boldface. BIC, Bayesian Information criterion;−2LL difference,−2 times Log-Likelihood difference between a N class solution and N-1 class solution; BLRT, Bootstrapped Likelihood Ratio Test; LMR-A, Lo-Mendell- Rubin Adjusted likelihood ratio test*.

**Figure 1:**
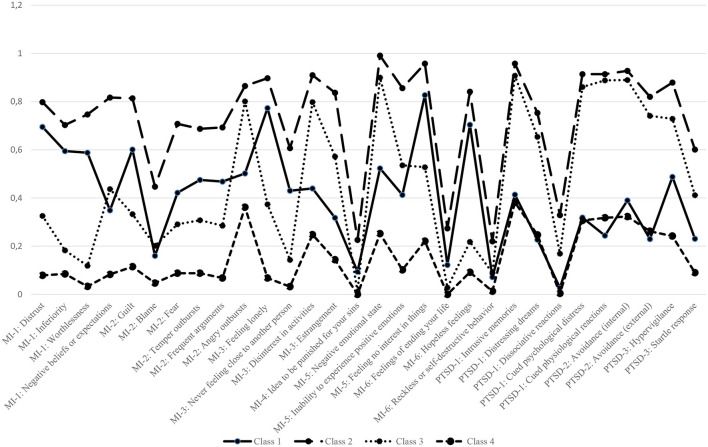
Symptom endorsement probability for the four-class solution. MI, moral injury; PTSD, post–traumatic stress disorder. *For full description of the items; see Table 2.

According to the model fit indices, all solutions up to six classes were possible optimal solutions. The LMR-A yielded a non-significant *p*-value (12) for the seven-class solution. Therefore, solutions with seven classes or more were not considered. All BLRT *p*-values were significant. Log-likelihood values increased and BIC values decreased substantially when moving from one- to two- and then to three-class solutions before flattening out, indicating diminishing gain in log-likelihood and BIC between the three-, four-, five-, and six-class solutions. Entropy remained quite similar over the various models with values >0.84, with the four-class solution showing the best entropy value (89). In the three-class solution there was a clear distinction between a severe class with high symptom endorsement on almost all items and a moderate class with overall low scores, and there was also a class with low scores on PTSD and varying scores for MI in which not all of the six MI components were met. The five-class solution did not result in clearly defined classes because two classes were interpretatively similar to one another (for graphs of the three- and five-class solutions, please see Supplementary Figures S1, S2). The four-class solution appeared to be the most meaningful, parsimonious, and best-fitting model. Most decisive was that this solution had the best interpretability. Figure 1 shows the symptom endorsement probability for the four-class solution, with the items operationalizing the elements of MI first, followed by those for PTSD.

Using a probability >0.5 as a cut-off value for symptom endorsement, we identified the following classes: (1) a *MI* class, with high symptom endorsement on most items representing MI components and low scores on the core items representing PTSD (*n* = 192; 11.27%); (2) a *MI-PTSD* class with high symptom endorsement on most items (*n* = 565; 33.18%); (3) a *PTSD* class, with low symptom endorsement on MI items and high symptom endorsement on the PTSD items (*n* = 644; 37.82%); and (4) a *Neither MI-nor PTSD* class with low symptom endorsement on all items (*n* = 302; 17.73%).The total occurrence of participants who met a MI symptom profile either with or without PTSD was 44.45% (*n* = 757).

Notably, four items had low (<0.5) symptom endorsement in all four classes: CAPS item B3 *Dissociative reactions*, one of the five items for the PTSD dimension Intrusions (0.33); BSI item 34 *The idea that you should be punished for your sins*, which was the only item representing the MI-4 dimension Failure to forgive or experience of self-condemnation (0.23); and BSI item 9 *Thoughts of ending your life* (0.27) and CAPS item E2 *Reckless or self-destructive behavior* (0.22), two of the three items representing the MI-6 dimension Self-harming and self-handicapping behaviors and demoralization. As can be seen in Table 2, these symptoms showed low endorsement rates in the total sample compared to all other symptoms: 17.7% for CAPS item B3 (*n* = 300); 9.1% for BSI item 34 (*n* = 155); 11.6% for BSI item 9 (*n* = 198) and 11.8% for CAPS item E2 (*n* = 199).

### Characterization of Class Membership

Table 4 presents the descriptive statistics of the variables age, gender, professional background, PTSD severity (CAPS-5), and psychopathology severity (BSI) for each of the four classes separately.

**Table 4:** Descriptive statistics of the variables within the four-class solution.

	**MI class (*****n*** **=** **192; 11.27%)**			**MI-PTSD class (*****n*** **=** **565; 33.18%)**			**PTSD class (*****n*** **=** **644; 37.82%)**			**Neither MI-nor PTSD class (*****n*** **=** **302; 17.73%)**		
	***n* (%)**	**M**	**SD**	***n* (%)**	**M**	**SD**	***n* (%)**	**M**	**SD**	***n* (%)**	**M**	**SD**
Variables	
Age	191	45.43	10.39	564	45.43	10.36	644	45.78	10.57	302	44.95	12.04
Gender	
Male	151 (79.1)			415 (73.6)			460 (71.4)			238 (78.8)		
Female	40 (20.9)			149 (26.4)			184 (28.6)			64 (21.2)		
Professional background	
Police force	179 (93.2)			476 (84.2)			585 (90.8)			291 (96.4)		
Military veterans	13 (6.8)			89 (15.8)			59 (9.2)			11 (3.6)		
PTSD severity (CAPS-5)	185	16.06	8.15	562	41.80	9.17	638	31.97	7.82	300	11.16	6.76
Psychopathology severity (BSI)	192	1.67	0.50	563	2.22	0.54	644	1.19	0.43	302	0.63	0.33

*MI, moral injury; PTSD, post-traumatic stress disorder; CAPS-5, Clinician-Administered PTSD Scale for DSM-5; BSI, Brief Symptom Inventory*.

The PTSD class was the largest class. Most police officers endorsed the PTSD class, whereas most military veterans endorsed the combined PTSD-MI class. Participants in the combined PTSD-MI class reported the most severe symptoms of PTSD and comorbid psychopathology.

Results of the multinomial logistic regression analyses are shown in Table 5.

**Table 5:** Results of the multinomial regression analysis of the four classes and the variables age, gender, PTSD severity, and psychopathology severity.

**Reference: MI class**	**PTSD class**			**MI-PTSD class**			**Neither MI-nor PTSD class**					
**Variables**	**B**	**SE**	**CI**	**Two-tailed *p*-value**	**B**	**SE**	**CI**	**Two-tailed *p*-value**	**B**	**SE**	**CI**	**Two-tailed *p*-value**
Age	0.090	0.094	−0.094 to 0.274	0.339	0.052	0.094	−0.132 to 0.236	0.580	−0.058	0.108	−0.270 to 0.154	0.591
Gender	0.552^*^	0.238	0.086 to 1.018	0.020	0.503^*^	0.240	0.033 to 0.973	0.036	−0.026	0.263	−0.541 to 0.489	0.923
PTSD severity (CAPS-5)	5.540^*^	0.480	4.599 to 6.481	0.000	7.971^*^	0.514	6.964 to 8.978	0.000	−1.268^*^	0.216	−1.691 to −0.845	0.000
Psychopathology severity (BSI)	−2.072^*^	0.195	−2.454 to −1.690	0.000	1.895^*^	0.207	1.489 to 2.301	0.000	−5.378^*^	0.319	−6.003 to 4.753	0.000

*MI, moral injury; PTSD, post-traumatic stress disorder; CAPS-5, Clinician-administered PTSD Scale for DSM-5; BSI, Brief Symptom Inventory*.

* *p < 0.05; B, log odd; SE, standard error; CI, 95% confidence Interval of regression coefficient B*.

The B coefficients (log odds) indicate how much more or less likely it becomes to be in the MI class (reference group) relative to the other classes with every unit increase in the covariate.

*Age* did not differ significantly between the classes. *Gender* differentiated significantly between the classes: women were more likely to endorse the MI-PTSD class and the PTSD class compared to the MI class. Gender did not differentiate between the other classes. *PTSD severity* also differentiated significantly between the classes: participants with higher levels of PTSD severity were more likely to endorse the MI-PTSD class (*M* = 41.80) and the PTSD class (*M* = 31.97) compared to the Neither MI-nor PTSD class (*M* = 11.16) or the MI class (*M* = 16.06). Finally, self-reported comorbid *psychopathology severity* differentiated significantly between the classes: participants reporting more severe comorbid psychopathology were more likely to endorse the MI-PTSD class (*M* = 2.22) and the MI class (*M* = 1.67) compared to the PTSD class (*M* = 1.19) and the Neither MI-nor PTSD class (*M* = 0.63).

In summary, the MI class was associated with male gender and lower PTSD severity. The combined MI-PTSD class consisted of patients with the highest PTSD and highest psychopathology severity. Military veterans were mostly represented in the combined MI-PTSD class and police officers were mostly represented in the PTSD class.

## Discussion

We conducted a latent class analysis of MI and PTSD in a sample of 1,703 trauma-exposed, treatment-seeking police officers and military veterans. We identified four classes of patients: a MI class (*n* = 192; 11.27%), a MI-PTSD class (*n* = 565; 33.18%), a PTSD class (*n* = 644; 37.82%), and a Neither MI-nor PTSD class (*n* = 302; 17.73%). The identification of classes characterized by high MI reflects findings of three latent profile analyses (LPA) of MI in military veterans (43–45). These previous studies identified two groups that were, respectively, high and low in MI plus complex PTSD (43), two MI groups characterized by psychological distress and spiritual distress, respectively, as well as a non-distressed group (44), and a high symptoms group, lower symptoms group, and potential MI group (45). Altogether these results confirm that MI is a prominent form of symptomatology amongst police officers and veterans exposed to profession-related trauma.

In our study, the group with a symptom profile of MI with or without PTSD is substantial (44.45%), in line with an earlier study of United States active duty military personnel that found a rate of 52% with high scores on at least four MI symptoms (14). Research of the prevalence of MI has focused primarily on exposure to PMIEs rather than on MI symptomatology. In the previously mentioned LPA's of MI in military veterans, the high MI distress group was 80.3% (43), the psychological MI group around 74% (44), and the potential MI group 22.2% (45). Most likely the definition of MI and the consequent selection of items influenced the prevalence of MI in different groups. In a previous study of Dutch military veterans, a quarter were found to experience feelings of shame, guilt, depression and anger (25). However, participants in this study were non-treatment-seeking, which may explain differences in prevalence.

Our finding of a separate MI class is also in line with other research in which PTSD and MI are defined as distinct constructs that often occur together (e.g., (21, 46, 47)), but that can also occur separately (24). The type of traumatic experience leads to a fear-based response during the event (e.g., “I will get hurt,” “I am going to die“) and/or a self-referential response after the event (e.g., “It is my fault,” “I am a failure”). The first is considered the “classic PTSD” with hyperarousal as one of its main symptoms and anxiety being mainly physiological. The latter response is associated with MI and is more related to existential fears (1) and perceived moral conflict (24, 46, 48). Farnsworth et al. (24) and Barnes et al. (48) advocate for clarifying the index trauma type that has evoked the most symptoms, as a potential indicator to distinguish between PTSD and MI.

We found demographic and clinical differences between the subgroups. The MI-PTSD class consisted mostly of veterans and the PTSD class was mostly made up of police officers. This unequal distribution across different classes reflects the fact that these two groups were not equally matched regarding symptom severity. The police officers showed a much wider variation in symptom severity, ranging from low to severe, compared to the veterans who mainly reported severe symptoms. This variation might be explained by different factors. First, all data of military veterans in this study were from ARQ Centrum'45, a highly specialized institute for psychotrauma, while the data from ARQ Diagnostic Centrum were limited to police officers, some of whom would not be referred for further treatment. Second, actual differences may exist between police officers and military veterans concerning PMIE exposure and subsequent MI, with military veterans potentially being exposed at a younger age (see (15, 17)) as well as potentially more frequently to traumatic events in childhood (49, 50). Further research is needed to examine if such differences between these populations indeed exist.

In contrast to the findings of Mantri et al. (15, 17) and LaFrance et al. (18), age did not differentiate between the four classes. However, there were significant differences between the classes in terms of gender distribution, with the MI classes consisting mostly of men. This echoes previous research in which PMIE exposure and functional impairment were found to differ between men and women (8). Last, significant differences were found between the classes in PTSD severity and comorbid psychopathology severity. In the MI class PTSD severity was low. In the MI-PTSD class participants showed the highest PTSD severity and psychopathology severity, reflecting a high level of suffering in general in this group of participants. These findings may partly be explained by item overlap in different steps of the analysis, given that some CAPS-5-items and BSI-items were used both as items in the LCA and as predictors. We therefore checked for possible interference and used separate models in the multinomial regression models. Findings are in line with another study that found MI scores to correlate with higher symptom severity of comorbid PTSD and major depressive disorder (44).

Four items had low symptom endorsement in all four classes: *Dissociative reactions, the idea that you should be punished for your sins, Thoughts of ending your life*, and *Reckless or self-destructive behavior*. Three of these items (except for dissociative reactions) were intended to measure MI. The low symptom endorsement suggests that in our sample, these items appeared less relevant to the MI construct. Given that The Netherlands are relatively secularized compared to the United States, “the idea that you should be punished for your sins” might be an item that appeals less to a Dutch sample. In a systematic review, transgressive acts were shown to be associated with a small but significantly increased risk of suicidality, but the overall incidence of suicidality was low (7). In another review, attempted suicide was associated with spiritual factors, including violation of own beliefs, rejected previously held religious beliefs, spiritual distress, and feeling unforgivable (21). Thoughts of ending your life might be relatively low in our sample either because of issues of secularization or because the sample consisted mainly of patients referred for outpatient treatment, i.e., who did not need hospitalization for suicidal levels.

### Strengths and Limitations

This is the first study of MI symptom profiles in a Dutch sample of trauma-exposed, treatment-seeking police officers and military veterans. Although there is a significant body of research on the concept of MI, prevalence studies are sparse and use different conceptualizations and measurements. Our study is the first LCA to build on the original conceptual framework for MI (1). The prevalence of MI symptoms has received relatively less attention than that of PMIEs. Studies of MI in police officers are especially rare (21, 51), and studies of MI in Dutch military veterans have been limited to non-treatment-seeking participants (5). Our study shows that the MI construct is relevant to Dutch police officers and military veterans seeking help for their trauma-related mental health problems. Sample size was high, involving a heterogeneous group of participants with a wide variety of symptoms and symptom severity.

A primary limitation of our study is that no data were available about specific transgressions and moral stressors. We used the A-criterion of PTSD as defined in the DSM-5 (19) as an inclusion criterion. Description of the A-criterion is limited to experiencing, witnessing, learning about or being exposed to aversive details of actual or threatened death, serious injury and sexual violence. Consequently, it is insufficiently indicative of whether these are events “in which a person perpetrates, fails to prevent, bears witness to, or learns about acts that transgress deeply held moral beliefs and expectations” ((1), p. 700). While we considered including the A-criterion in our analyses, we decided against this as the literature provided insufficient guidance for hypotheses.

Another limitation was that we used an existing dataset that did not contain instruments specifically designed for assessing MI. At the time of data inclusion, no reliably translated and validated Dutch-language MI measurement was available. We are now in the process of validating two reliably translated instruments in a sample of military veterans. In the current study, MI items were carefully selected from the CAPS-5 and BSI by independent assessors to match the MI framework. However, not all items may have exactly fit. Unlike the CAPS-5, the BSI does not inquire about trauma-relatedness of the symptoms, which is another limitation of using this instrument.

### Conclusion and Recommendations

This study indicates that trauma-exposed, treatment seeking police officers and military veterans can suffer from symptoms that could be labeled as MI. Given the relevance of MI to those groups, we recommend routinely screening for MI using instruments such as the Moral Injury Events Scale (MIES) (52), which assesses both exposure and distress, and/or the Expressions of Moral Injury Scale-Military Version (EMIS-M) (53) and Moral Injury Symptom Scale-Military Version (MISS-M) (13), which both measure distress. In addition, instruments assessing MI outcomes in police officers or, more generally, in first responders, are needed.

As MI and PTSD may occur separately as well as together among treatment-seeking police officers and military veterans, it may be concluded that trauma-focused interventions may be insufficient in some individuals and that in those cases, adding interventions that focus on MI may be warranted. While PTSD and depression related to moral injurious events may be effectively treated with trauma-focused treatment (23), other symptoms may remain that may respond to interventions designed especially for MI, such as Adaptive Disclosure (54), Trauma-Acceptance and Commitment Therapy for Moral Injury (ACT-MI) (55) and Trauma-Informed Guilt Reduction Therapy (56).

In order to further the study of the prevalence of MI in PMIE-exposed individuals, several factors are of importance. First, a consensus definition of MI is needed. Currently, definitions and consequently, assessments differ, resulting in differences in prevalence that may be unrelated to population and exposure. Second, most studies of MI have been conducted in military personnel. Studies of MI in police officers and other first responders are needed given their likely high exposure to PMIEs. Third, in order to do so, diagnostic instruments need to be developed and tested in those specific populations.

In conclusion, MI appears to be prevalent in treatment-seeking police officers and military veterans, which may need to be taken into account when tailoring treatment.

## Data Availability Statement

Data were collected primarily for clinical purposes and are not deposited in a community-recognized repository because participants have not provided informed consent for sharing data outside of the institute. Requests to access these datasets should be directed to b.mensink@arq.org.

## Author Contributions

BM, AS, and FH: contributions to the conception or design of the work. BM and NA: acquisition and analysis. BM, AS, FH, and NA: interpretation of data for the work and drafting the work or revising it critically for important intellectual content. All authors have approved for publication of the content and have agreed to be accountable for all aspects of the work.

## Conflict of Interest

The authors declare that the research was conducted in the absence of any commercial or financial relationships that could be construed as a potential conflict of interest.

## Publisher's Note

All claims expressed in this article are solely those of the authors and do not necessarily represent those of their affiliated organizations, or those of the publisher, the editors and the reviewers. Any product that may be evaluated in this article, or claim that may be made by its manufacturer, is not guaranteed or endorsed by the publisher.
