# Supplementary material for: Moral Injury in Trauma-Exposed, Treatment-Seeking Police Officers and Military Veterans: Latent Class Analysis
Source: Front Psychiatry. 2022 Jul 11;13:904659. doi: 10.3389/fpsyt.2022.904659 (PMC9311258; doi:10.3389/fpsyt.2022.904659)
Supplement: Supplementary file 1 [file Data_Sheet_1.docx]

**Supplementary figures**

Figure S1: Symptom endorsement probability for the three-class solution

MI: moral injury, PTSD: posttraumatic stress disorder.
*For full description of the items, see table 2.

Figure S2: Symptom endorsement probability for the five-class solution

MI: moral injury, PTSD: posttraumatic stress disorder.
*For full description of the items, see table 2.
